# Genome-Wide Identification and Characterization of the JAZ Gene Family in *Malus sieversii*

**DOI:** 10.3390/genes17070742

**Published:** 2026-06-26

**Authors:** Xumin Wang, Baofeng Hao, Chao Zhang, Yue Yao, Yongjie Wu, Jintao Xu

**Affiliations:** Changli Institute of Pomology, Hebei Academy of Agriculture and Forestry Sciences, Qinhuangdao 066600, China; minr@nwafu.edu.cn (X.W.); haobf1973@163.com (B.H.); 18838933981@163.com (C.Z.); 18931353275@163.com (Y.Y.); wuyongjie007@163.com (Y.W.)

**Keywords:** jasmonic acid, ethylene, physical interaction, softening

## Abstract

Background: *Malus sieversii*, the wild ancestor of cultivated apples, possesses high stress tolerance and rich nutritional value but suffers from low fruit firmness. Jasmonate-ZIM (JAZ) domain proteins are key repressors of jasmonic acid (JA) signaling, yet their roles in fruit softening remain largely unexplored, especially in *M. sieversii*. Methods and results: In this study, we performed a genome-wide identification of the JAZ gene family in *M. sieversii* and characterized their structural features, phylogenetic relationships, chromosomal distribution, promoter cis-elements, expression patterns, and protein interactions. A total of 18 *MsiJAZ* genes were identified, which could be classified into six subfamilies. Most members are predicted to localize in the nucleus, while three are also potentially targeted to chloroplasts. The *MsiJAZ* genes are unevenly distributed across ten chromosomes and are enriched in light-, ABA-, and drought-responsive cis-elements. Expression analysis under storage and 1-MCP treatments revealed both shared and divergent responses among selected *MsiJAZ* genes. Notably, MsiJAZ1 was significantly repressed by storage but induced by 1-MCP and physically interacted with MsiPUB24 (PLANT U-BOX 24), an E3 ubiquitin ligase involved in ethylene-mediated softening. These results suggest that MsiJAZ1 may participate in a novel CORONATINE INSENSITIVE 1 (COI1)-independent degradation pathway and mediate ethylene–JA crosstalk during fruit softening. Conclusions: This study provides a comprehensive characterization of the JAZ family in *M. sieversii* and lays a foundation for further functional studies on JA signaling in apple fruit softening.

## 1. Introduction

A narrowing of the genetic base, driven by inbreeding depression, has seriously constrained apple production and diminished the nutritional quality of modern cultivars. At the same time, *Malus sieversii* is now increasingly appreciated for its essential role in the domestication and genetic enhancement of apple [1,2]. Commonly known as “Xinjiang wild apple”, *M. sieversii* is largely confined to the Tianshan region in China, Kazakhstan, Uzbekistan and Kyrgyzstan [3,4]. As the wild ancestor of cultivated apples, it exhibits superior nutritive value and health-promoting properties, thereby positioning it as a valuable germplasm for enhancing both environmental resilience and flavonoid accumulation in cultivated apples [1,5]. However, compared with cultivated apples, *M. sieversii* has much lower fruit firmness [6]. Elucidation of the key genetic regulators of fruit softening in *M. sieversii* is therefore fundamental to its conservation and breeding application.

Jasmonates (JAs) comprise jasmonic acid (JA) and its oxylipin derivatives, which are lipid-based phytohormones that mediate plant defense against herbivores and pathogens, as well as responses to UV radiation, ozone, and other abiotic stresses [7,8,9,10]. In addition, JA triggers the accumulation of secondary metabolites such as alkaloids, anthocyanins, and terpenoids [10,11,12]. Additionally, MeJA regulates fruit development via modulation of endogenous JA levels [13,14]. Jasmonate-ZIM domain (JAZ) proteins act as key repressors in the JA signaling pathway by negatively regulating downstream responses [15]. Under low JA levels, JAZ transcriptional repressors interact with MYC2 and other transcription factors (TFs) to suppress the expression of early JA-responsive genes [16]. When JA levels increase, JAZ proteins undergo ubiquitination and are subsequently degraded by the 26S proteasome, thereby relieving the repression [16,17].

JAZ proteins belong to the TIFY subfamily and consist of two major domains: the TIFY domain and a C-terminal conserved region termed the Jas domain [18]. The Jas domain shares sequence similarity with the N-terminal portion of the CCT domain with a characteristic SLX2FX2KRX2RX5PY motif [19]. JAZ proteins can interact with various TFs, such as MYC, JAM, ICE, MYB, and TOE, to modulate specific downstream gene expression. This regulation contributes to diverse biological processes, including root elongation, leaf aging, insect defense, freezing stress adaptation, and floral initiation [20,21,22,23,24,25]. Recently, studies on apple have further elucidated how JAZ-mediated transcriptional regulation regulates critical agronomic traits [26]. The JAZ-BBX37-ICE1-CBF module regulates JA-mediated cold tolerance [27]. MdABI4 interacts with MdICE1 and JAZ proteins to regulate abscisic acid-mediated cold tolerance [28]. The MdJAZ2–MdSINA11 module regulates both JA signaling and JA-induced anthocyanin accumulation [29]. The JAZ1–TRB1–MYB9 complex also mediates JA-induced anthocyanin accumulation [11]. Meanwhile, MdJAZ2 interacts with MdWER to regulate light-induced anthocyanin accumulation [30]. MdZFP7 coordinates JA and GA signaling through interactions with MdJAZ2 and MdRGL3a to regulate anthocyanin accumulation [31]. The stability of MdJAZ2 is modulated by the MdBT2 protein, which thereby negatively regulates JA-triggered leaf senescence [32]. MdERF4 mediates crosstalk between ethylene (Eth) and JA signaling pathways by interacting with JAZ proteins, thereby influencing fruit ripening [33]. Compared with its well-established roles in anthocyanin accumulation and other processes, the involvement of JAZ proteins in fruit softening remains poorly understood in apple.

*M. sieversii* is valued for its strong stress tolerance and rich nutrient composition, yet the contribution of its *JAZ* genes to fruit softening remains unclear [19]. Here, we systematically mined the JAZ family in this wild apple species by leveraging the high-quality *M. domestica* genome and the JAZ classification scheme from *A. thaliana* [34,35]. A total of 18 JAZ genes were uncovered and characterized with respect to their structural organization, conserved domains, cellular compartment predictions, chromosomal distribution, phylogenetic placement, and promoter regulatory features. MsiJAZ1 was further examined through RT-qPCR and protein interaction assays. This work provides a valuable resource for future studies on fruit quality and stress adaptation in apple.

## 2. Materials and Methods

### 2.1. Identification of Jaz Gene Family Members in M. sieversii

A BLASTP search was performed via TBtools (v2.056) [36] against the *M. sieversii* proteome using *A. thaliana* JAZ protein sequences as queries, with E-value < 1 × 10^−5^ and ≥60% coverage as cutoffs. Redundant hits were discarded, and the remaining candidates were aligned with DNAMAN to verify their uniqueness. Domain architecture was initially explored with the NCBI CDD database (https://www.ncbi.nlm.nih.gov/Structure/cdd/wrpsb.cgi, accessed on 1 April 2026) and later corroborated using the PFAM database (http://pfam.xfam.org/, accessed on 1 April 2026) and the SMART database (https://smart.embl.de/, accessed on 1 April 2026), with an emphasis on the Jas domain. This approach resulted in the identification of the complete JAZ gene set in *M. sieversii*. Supplementary Table S1 summarizes the genomic information for *A. thaliana*, *M. domestica*, *M. sieversii*, *O. sativa*, *P. betulifolia*, *S. lycopersicum*, and *V. vinifera*.

### 2.2. Phylogenetic Analysis of the Jaz in M. sieversii

Alignment of sequences was conducted with ClustalW (MEGA X) [37]. The Neighbor-Joining (NJ) method was applied to reconstruct the phylogenetic tree, and the reliability of internal branches was evaluated by bootstrap analysis based on 1000 resamplings.

### 2.3. Chromosomal Localization of Msijazs

The chromosomal positions of all *M. sieversii* JAZ genes were determined from the retrieved genomic data, and a chromosome localization map was subsequently generated.

### 2.4. Analysis of Phylogenetic, Conserved Motifs and Gene Structure of MsiJAZs

GSDS 2.0 (http://gsds.cbi.pku.edu.cn/, accessed on 1 April 2026) and MEME (https://meme-suite.org/meme/, accessed on 1 April 2026) were applied for gene structure and conserved motif analysis of the *M. sieversii* JAZ family. *M. sieversii* JAZ homologs were retrieved via homology search against the CDS database using *A. thaliana* JAZ sequences as queries. A Maximum Likelihood tree (1000 bootstrap replicates) was constructed from the combined JAZ sequences of both species using MEGA X and visualized with iTOL (https://itol.embl.de/, accessed on 1 April 2026).

### 2.5. Cis Acting Element Analysis

The 2 kb upstream regions of JAZ genes were extracted from the *M. sieversii* genome for cis-element prediction. PlantCARE (http://bioinformatics.psb.ugent.be/webtools/plantcare/html/, accessed on 1 April 2026) was used to identify putative regulatory motifs. Excel 16.0 was applied for data handling, and TBtools (v2.056) [36] for visualization.

### 2.6. Quantitative Real-Time PCR (RT-qPCR) Assay

Following previously described methods [38], 60 fruits of uniform size and free from visible defects were collected at commercial harvest and randomly divided into three groups. One group was sampled immediately at harvest (designated as 0-Pre). The remaining fruits were divided into two subsets: one was directly stored at room temperature (24 °C) for 15 days, while the other was first treated with 1-MCP (Fresh Doctor, Shenzhen, China) and then stored under the same conditions. At the end of the storage period, the untreated subset was designated as 15-Post, and the 1-MCP-treated subset was designated as 15-MCP. For 1-MCP treatment, the fruit was exposed to 1-MCP (1 μL/L) for 12 h at room temperature in a plastic crisper container. Samples were immediately snap-frozen in liquid nitrogen and then maintained at −80 °C. Gene-specific primers were designed via Primer3.0 and synthesized at Sangon Biotech (Shanghai, China). *Actin* was used as the reference gene. RNA was extracted with the RNA Plant Kit (Transgene, Beijing, China) and reverse-transcribed using the PrimeScript RT Reagent Kit (Takara, Shiga, Japan) under the manufacturer’s conditions. Three biological replicates were performed for each condition. Relative expression was calculated by the 2^−ΔΔCT^ method. Supplementary Table S2 contains all RT-qPCR primer sequences.

### 2.7. Yeast Two-Hybrid (Y2H) Assay

Fusion expression constructs were produced by inserting the amplified CDS of *MsiJAZ1* and *MsiPUB24* from *M. sieversii* cDNA into pGADT7 and pGBKT7, respectively. The resultant BD-MsiPUB24 and AD-MsiJAZ1 plasmids, along with empty vector controls, were co-transformed into yeast competent cells. Initial selection was performed on SD/−Trp/−Leu double dropout medium at 30 °C for 4–5 days. To confirm interactions, six colonies per transformation were replated onto SD/−Trp/−Leu/−His/−Ade quadruple dropout medium and incubated for another 3–5 days.

### 2.8. Luciferase Assay

Using *M. sieversii* cDNA as template, the open reading frames of *MsiPUB24* and *MsiJAZ1* were amplified and individually fused into pCAMBIA1300-nLUC and pCAMBIA1300-cLUC vectors. The recombinant plasmids were delivered into *Nicotiana benthamiana* leaf tissues via *Agrobacterium*-mediated infiltration for transient expression. A 100 mM D-luciferin potassium salt stock solution was prepared by dissolving 25 mg of the substrate in 0.7852 mL of sterile water. This concentrate was then diluted with sterile water to a final concentration of 1–5 mM immediately before use, and the resulting solution was spotted evenly onto the infiltrated leaf regions. Following a 5 min dark incubation, luminescence was recorded using a Tanon in vivo plant imaging system.

## 3. Result

### 3.1. Identification and Phylogenetic Analysis of Members of the JAZ Gene Family in M. sieversii

A total of 18 JAZ proteins were identified in the *M. sieversii* genome through a combination of BLASTP (2.2.28) and HMMER (3.2.1) searches followed by manual curation. Alignment of these MsiJAZ protein sequences revealed that they possess two characteristic domains, TIFY and Jas, which are typical of the JAZ family. Notably, MsiJAZ15 lacks the TIFY domain, and accordingly, its predicted three-dimensional structure differs significantly from those of the other members, primarily in its reduced β-sheet (Supplementary Figure S1).

Physicochemical properties of the 18 encoded proteins were examined using the ProtParam tool, revealing marked differences among the JAZ protein sequences (Supplementary Table S3). These proteins ranged in length from 110 to 395 amino acids, with the majority falling between 200 and 300 aa. Their molecular weights spanned 12.14–41.47 kDa. The isoelectric points (pI) ranged from 5.96 to 9.43; only three had a pI below 7 (acidic), whereas the other fifteen were basic. This diversity in pI values may reflect functional divergence among JAZ proteins in mediating protein–protein interactions or subcellular localization. The average hydrophilicity values of these proteins ranged from −0.817 to −0.230, suggesting that all JAZ family members are hydrophilic in nature. Subcellular localization analysis revealed that 15 members were predicted to be localized in the nucleus, which is consistent with their primary role as repressors of JA signaling—associating with transcription factors and regulating downstream gene expression. Notably, subcellular localization predictions also indicated that three members (MsiJAZ2, 6, and 17) may reside in the chloroplast. These dual-localization findings raise the possibility that certain JAZ proteins could be subject to post-translational modifications, engage in protein–protein interactions, or respond to signals, thereby allowing dynamic movement between chloroplast and nucleus to modulate their transcriptional activity.

**Figure 1:**
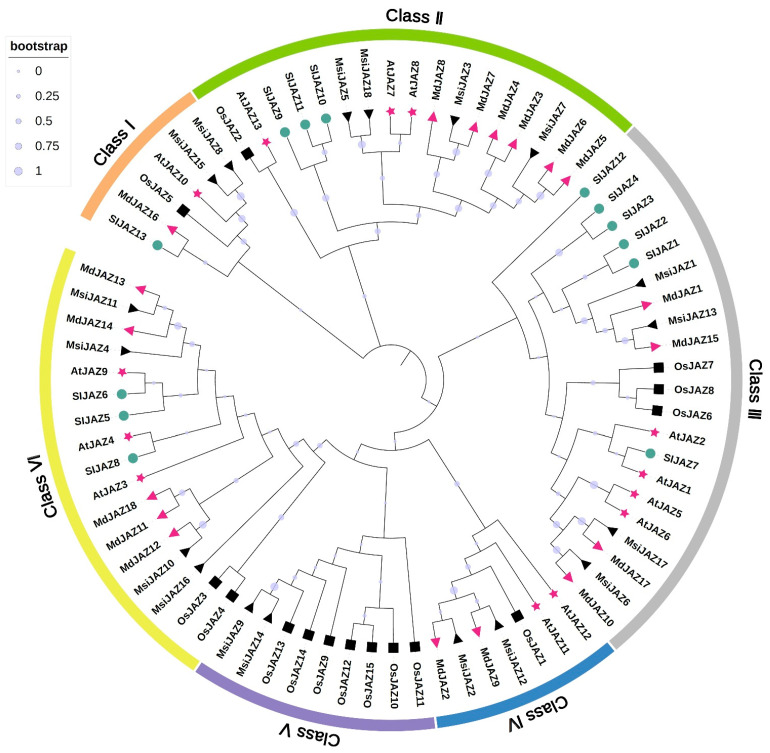
Systematic evolutionary analysis of JAZ family proteins. Neighbor-joining tree representing phylogenetic relationships among *JAZ* genes from *A. thaliana*, *M. sieversii*, *M. domestica, O. sativa,* and *S. lycopersicum*.

Additionally, a phylogenetic tree was built using the full-length amino acid sequences of JAZ proteins from 18 *M. domestica* var. ‘Golden Delicious’, 13 *A. thaliana*, 15 *O. sativa*, 13 *S. lycopersicum*, and the 18 *M. sieversii* proteins identified in this study (Figure 1). Phylogenetic analysis placed the 18 MsiJAZ proteins into six subfamilies. The Class I subfamily comprised 2 members orthologous to the functionally characterized *A. thaliana* gene *JAZ10*, with *MsiJAZ8* and *MsiJAZ15* uniquely containing Motif 8. The Class VI subfamily consisted of four *M. sieversii* members, all of which specifically possessed Motif 7; additionally, *MsiJAZ10* and *MsiJAZ16* also uniquely harbored Motif 9. In *M. domestica*, 1 Class I, 6 Class II, 4 Class III, 2 Class IV, 0 Class V, and 5 Class VI members were identified, which differ from the numbers in *M. sieversii*. Although the total number of JAZ genes is comparable between the two germplasms, the distinct distribution across subfamilies suggests a possible functional diversification of certain subfamilies in *M. domestica*.

### 3.2. Analysis of MsiJAZ Gene Structure, Motifs, and Domains

**Figure 2:**
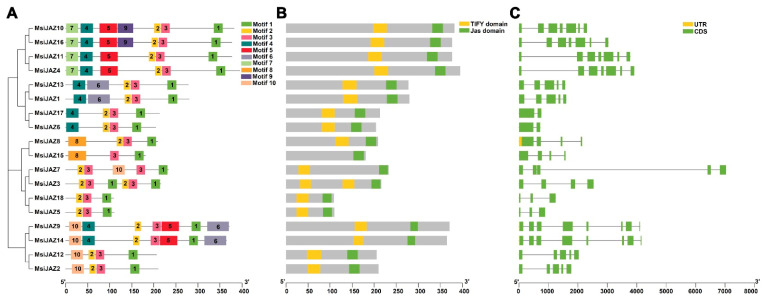
Structural overview of MsiJAZ proteins and genes. (**A**) Conserved motifs (Motifs 1–10) identified via MEME are mapped onto the protein sequences. (**B**) Domain architecture. (**C**) Genomic organization of CDS and UTR regions.

Gene structure analysis of *M. sieversii* JAZ genes revealed considerable variation among most members, with exon numbers ranging from 2 to 8 and intron numbers from 1 to 7; the majority had 5 exons and 4 introns. Furthermore, only MsiJAZ8 was found to contain UTR sequences (Figure 2C).

We subsequently analyzed the conserved motifs of the MsiJAZ proteins and identified 10 motifs (designated Motif 1–10), ranging in length from 15 to 50 amino acids. Annotation using Pfam and SMART revealed that Motif 2 and Motif 1 correspond to the conserved TIFY and Jas domains, respectively (Supplementary Figure S2). As shown in Figure 2A, all MsiJAZ proteins contain both Motif 1 and Motif 3. Among them, MsiJAZ9, 10, 14, and 16 harbor the largest number of motifs (seven each), whereas MsiJAZ5, 15, and 18 contain the fewest (only three). Phylogenetic analysis indicated that evolutionarily close members generally share similar motif compositions. Moreover, most MsiJAZ proteins contain Motifs 1–4. Overall, the MsiJAZ proteins exhibit a high degree of conservation. Conserved domain analysis (Figure 2B) demonstrated that all MsiJAZ proteins possess the Jas domain, with MsiJAZ15 containing only this domain.

### 3.3. Chromosome Mapping of MsiJAZs

Chromosomal locations of the *MsiJAZ* genes were retrieved from the *M. sieversii* genome database, and a distribution map was generated using TBtools (Supplementary Figure S3). The 18 *MsiJAZ* genes were unevenly distributed across ten chromosomes. Specifically, chromosomes 5 (*MsiJAZ3*), 6 (*MsiJAZ4*), 10 (*MsiJAZ7*), and 14 (*MsiJAZ11*) each harbored a single copy. Chromosomes 2, 9, 15, and 17 each contained two copies, while chromosomes 13 and 16 each contained three copies. Collectively, the *MsiJAZ* gene family exhibits a single-copy distribution throughout the apple genome.

### 3.4. Analysis of Cis-Acting Elements

**Figure 3:**
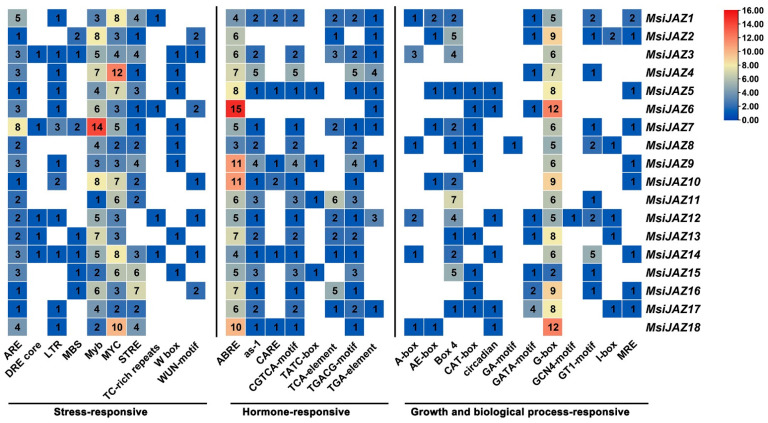
*Cis*-acting elements in the promoter regions of *MsiJAZs*. Element categories are listed at the bottom, with the corresponding numbers representing the count of each type.

To survey potential regulatory motifs, the 2 kb promoter regions upstream of the *JAZ* genes in *M. sieversii* were subjected to PlantCARE analysis (Figure 3). A total of 30 distinct cis-elements were identified in the promoters of the 18 *MsiJAZ* genes, falling into three functional categories: stress-related (Myb, MYC, ARE, LTR, and STRE), hormone-responsive (ABRE, as-1, and TGACG-motif), and growth/development-associated (G-Box, Box 4, and GT1-motif). Across the *MsiJAZ* promoters, ABRE and G-box motifs—linked to ABA and light signaling—were particularly enriched. In terms of individual genes, *MsiJAZ6* carries 15 ABRE and 12 G-box copies, whereas *MsiJAZ7* contains 14 MYB elements. Such variation in cis-element composition points to functional diversification of *MsiJAZs* in mediating hormonal and environmental signals.

### 3.5. Collinearity Analysis of MsiJAZs

**Figure 4:**
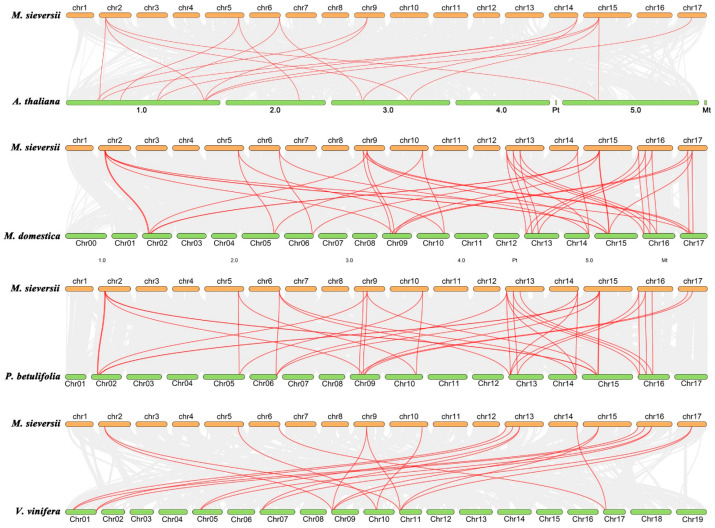
Collinear analysis of *M. sieversii*, *A. thaliana*, *M. domestica*, *P. betulifolia*, and *V. vinifera JAZ* family genes. Red lines indicate syntenic gene pairs.

To explore the evolutionary trajectory of the *JAZ* gene family in *M. sieversii*, we carried out comparative synteny analyses involving four representative species: *A. thaliana*, *M. domestica*, *Pyrus betulifolia*, and *Vitis vinifera* (Figure 4). A separate collinearity analysis integrating *M. sieversii*, *V. vinifera*, and *P. betulifolia* was performed to assess evolutionary conservation and divergence, integrating closely allied Rosaceae lineages and a basal eudicot with an ancestral genome. The count of syntenic gene pairs exhibited marked variation across species: the fewest were found between *M. sieversii* and *V. vinifera* (20 pairs), while the highest was with *M. domestica* (43 pairs), indicating a closer evolutionary relationship between the *JAZ* families of *M. sieversii* and *P. betulifolia*. A similar syntenic count was observed with *P. betulifolia* (42 pairs), reflecting conserved synteny within the *Malus* lineage. By contrast, only 24 syntenic pairs were identified with *A. thaliana*, comparable to the number with *V. vinifera*. Compared with *A. thaliana*, 16 of the 18 *MsiJAZ* genes showed syntenic relationships, whereas two (*MsiJAZ5* and *MsiJAZ7*) did not. When compared with *M. domestica*, all *MsiJAZ* genes exhibited signs of expansion, suggesting that most members underwent duplication events during the evolution of cultivated apples. Taken together, the synteny-based analyses suggest that the JAZ family in *M. sieversii* has undergone both purifying selection and lineage-restricted diversification, while retaining its core functional roles.

### 3.6. Expression Analysis of Six MsiJAZs

**Figure 5:**
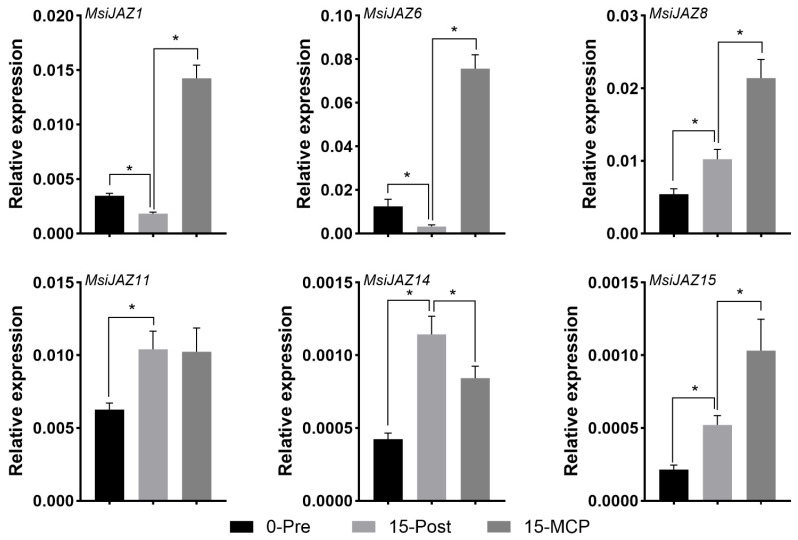
Transcript levels of six *MsiJAZs* following storage and 1-MCP treatment. Data are expressed as mean ± SD from three or more biological replicates. Asterisks denote statistically significant differences relative to the control (two-tailed paired Student’s *t*-test; *, *p* ≤ 0.05).

To identify candidate *JAZs* involved in apple fruit softening, we collected fruit at three stages: immediately after harvest (0 d, pre-climacteric, designated 0-Pre), after 15 d of room-temperature storage (post-climacteric, designated 15-Post), and following 1-MCP treatment (applied at commercial harvest followed by 15 d of room-temperature storage, designated 15-MCP). Based on the analysis of gene structure and cis-acting elements, six *MsiJAZs* were selected for expression analysis (Figure 5). *MsiJAZ1* and *MsiJAZ6* exhibited similar expression patterns: their transcript levels decreased following storage treatment, whereas 1-MCP treatment suppressed this decline and significantly elevated their expression. In contrast, the expression levels of *MsiJAZ8*, *MsiJAZ11*, *MsiJAZ14*, and *MsiJAZ15* increased after storage treatment. Similarly, under 1-MCP treatment, the expression of *MsiJAZ8* and *MsiJAZ15* continued to increase relative to storage alone, whereas *MsiJAZ14* showed a slight decrease, and *MsiJAZ11* exhibited no significant change. In conclusion, expression analysis of the six selected *MsiJAZs* revealed both similarities and divergences in their responses to storage and 1-MCP treatments, suggesting their potential involvement in phytohormone-mediated softening regulatory networks.

### 3.7. Physical Interaction Between MsiJAZ1 and MsiPUB24

**Figure 6:**
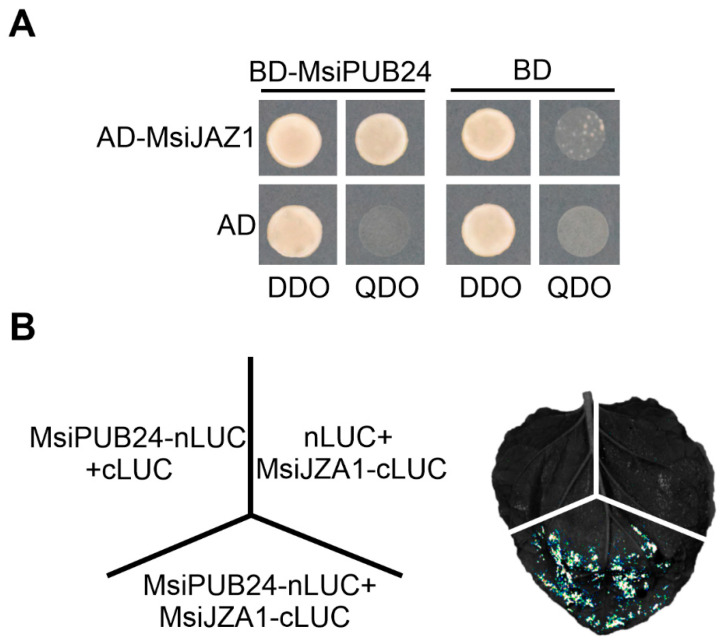
Verification of MsiJAZ1–MsiPUB24 interaction. (**A**) Y2H assay. (**B**) Split-LUC assay. DDO, SD/−Trp/−Leu; QDO, SD/−Trp/−Leu/−His/−Ade. Empty vectors (AD, BD, nLUC, cLUC) served as negative controls.

To test whether *MsiJAZ1* is involved in *MsiPUB24*-mediated signaling, we carried out yeast two-hybrid (Y2H) and split-luciferase complementation assays. MsiPUB24 was selected as a potential binding partner of MsiJAZ1 because of its key roles in ethylene-mediated softening regulation. The full-length coding sequences of *MsiPUB24* and *MsiJAZ1* were inserted into pGBKT7 and pGADT7, respectively. Co-expression of these constructs in Y2H Gold yeast cells allowed growth on SD/−Leu/−Trp/−His/−Ade medium, indicating that MsiPUB24 and MsiJAZ1 directly interact. This finding was corroborated by split-luciferase assays in *Nicotiana benthamiana* leaves: co-infiltration of nLUC-MsiPUB24 and cLUC-MsiJAZ1 generated robust luciferase signals (Figure 6). Collectively, these data demonstrate that MsiPUB24 and MsiJAZ1 physically associate both in vitro and in vivo.

## 4. Discussion

As a tertiary relict species and the wild ancestor of cultivated apples, *M. sieversii* harbors a wealth of genetic and phenotypic variation, far exceeding that found in modern apple cultivars, as a result of prolonged natural selection in the Tianshan Mountains. Its notable resilience to drought, chilling temperatures, nutrient-poor soils, and pathogen attack positions it as a valuable wild germplasm for breeding programs aimed at enhancing stress tolerance in apple. In addition, it displays unique morphological, reproductive, and adaptive characteristics that distinguish it from cultivated apples [1,2]. As a conserved family, *JAZ* has been characterized in various fields and horticultural crops, including tomato, maize, soybean, wheat, rice, strawberry, and wax apple [17,39,40]. Certain conserved TF families, such as ZFP and NAC, play essential roles in nutrient and quality metabolism during plant evolution [41,42]. However, many plant transcriptional regulators with known evolutionary importance still lack comprehensive functional characterization. Thus, further exploration of *JAZ* gene functions and underlying mechanisms is clearly warranted.

In this study, 18 MsiJAZ genes were identified in *M. sieversii*, equal to the number found in the cultivated apple cultivar ‘Golden Delicious’. However, the distribution of these genes among different subfamilies differed between the two genotypes, suggesting functional divergence possibly driven by their distinct growth environments. Notably, *MsiJAZ* genes are preferentially located at the distal ends of chromosomes. This non-random chromosomal distribution may be correlated with their conserved roles in phytohormone signaling and stress adaptation, potentially enabling rapid transcriptional responses to environmental stimuli (Supplementary Figure S3).

Phylogenetic analysis of five representative land plant species showed that *JAZ* genes split into six distinct subfamilies early in evolution (Figure 1). Sequence similarity, phylogenetic relationships, and intron positions further support this classification, which applies to wild *M. sieversii* and matches previous classifications in other land plants [43]. Gene duplication occurs through segmental duplication, random duplication, or retroposition [44]. Polyploidization results in large duplicated chromosomal segments in plants, with segmental duplication being the key driver of gene family expansion [45]. Synteny analysis suggested that some JAZ genes in *M. sieversii* have expanded over evolution (Figure 4).

All 18 MsiJAZ proteins were found to contain Motif 1, which was confirmed by structural analysis to reside within the JAZ family’s conserved domain. This indicates that Motif 1 is a highly conserved element essential for JAZ protein functions (Figure 2). The exon–intron organization of *JAZ* genes in *M. sieversii* is relatively complex, with only seven members having fewer than five exons—a pattern closely resembling that reported in alfalfa and wheat [40,46]. Genes with multiple introns can generate multiple protein isoforms via alternative splicing, potentially contributing to functional diversification and evolutionary adaptability. Future research using transcriptomic data from *M. domestica* or *M. sieversii* will be necessary to identify and validate possible splice variants of *JAZ* genes.

Cis-acting elements act as binding sites for transcription factors, helping plants adapt to environmental changes through transcriptional control. In *M. sieversii*, promoters of all 18 *MsiJAZ* genes contain light- and ABA-responsive motifs, as well as MYB binding sites linked to drought induction. A variety of hormone-responsive cis-elements, including auxin and jasmonic acid motifs, were also detected in their promoter regions (Figure 3). These observations imply that MsiJAZ proteins likely participate in responding to diverse environmental signals and may influence fruit development by regulating relevant phytohormone pathways.

*M. domestica* is closely related to *M. sieversii*, and its high-quality genome assembly and annotation provide a reliable reference for sequence alignment, gene discovery, and evolutionary analysis in this study [34]. The high syntenic pair numbers between *M. sieversii* and *M. domestica* (43) as well as with *P. betulifolia* (42) indicate strong conservation of the *JAZ* family within the Rosaceae lineage. In contrast, the much lower numbers with *V. vinifera* (20) and *A. thaliana* (24) suggest considerable divergence from more distantly related species. Thus, it is possible that the *JAZ* family in *M. sieversii* has been largely retained within Rosaceae, whereas gene loss and divergence may have taken place outside this family.

JA signaling, despite its functional diversity, converges on the F-box protein COI1. Upon JA-Ile perception, the SCF^COI1^ complex targets JAZ proteins for ubiquitination, marking them for proteasomal degradation via the 26S proteasome. This degradation relieves the repressive effect of JAZs on downstream transcription factors [10,47]. Nevertheless, hormone regulation mediated by the ubiquitination pathway is inherently complex. It has been reported that MdBT2 may function as a scaffold protein linking E3 ubiquitin ligases to target proteins, thereby facilitating their ubiquitination and degradation [48]. In GA signaling, DELLA proteins are not only targeted by SLY1 but also by multiple other substrate-recognition receptors [49]. Similarly, evidence indicates that JAZ proteins can be degraded independently of COI1, yet the full repertoire of such pathways and their biological significance remain largely unknown [50]. Herbivory-induced tomato PUB22 degrades non-COI1 JAZs via ubiquitination, promoting defense and JA signaling [51]. Seed-expressed Arabidopsis SKIP31 degrades JAZ6/11 independently of COI1, releasing ABI5 to activate seed maturation genes in *A. thaliana* [15]. In apples, ubiquitination is also implicated in the fruit softening process. Ethylene-activated E3 ubiquitin-like 1 (MdEAEL1) ubiquitinates and degrades the transcriptional repressor MdZFP3, thereby relieving the repression of cell wall degradation-related genes and promoting fruit softening [41]. MdNAC72 undergoes MdPUB24-mediated ubiquitination and degradation, a process strengthened by the ethylene-induced MdMAPK3 phosphorylation of MdNAC72, which relieves MdNAC72-mediated transcriptional repression of *MdPG1*, thereby promoting fruit softening during storage [42]. This study demonstrated that MsiJAZ1 physically associates with MsiPUB24 (Figure 6) and that most of the six *JAZ* genes examined were responsive to the treatments applied. Notably, *MsiJAZ1* was significantly repressed by storage but increased by 1-MCP (Figure 5). Thus, these results indicate that MsiJAZ1 may undergo MsiPUB24-mediated ubiquitination and degradation, and that MsiPUB24 might represent a novel COI1-independent pathway for JA signaling. Furthermore, MsiJAZ1 may be involved in the ethylene–JA crosstalk governing fruit softening during storage, providing a clue for further investigation of its function as a potential transcriptional regulator. Notably, future studies using overexpression and CRISPR/Cas9-mediated gene editing are still required to better define the biological functions of *MsiJAZ1*.

## 5. Conclusions

In this study, we performed a genome-wide identification and comprehensive characterization of the JAZ gene family in *Malus sieversii*, a wild apple species with high stress tolerance and rich nutritional value but with low fruit firmness. A total of 18 *MsiJAZ* genes were identified, which were classified into six subfamilies. *MsiJAZ* genes are unevenly distributed across ten chromosomes, and their promoters are enriched in light-, ABA-, and drought-responsive cis-elements, suggesting their potential involvement in multiple stress and hormone signaling pathways. Expression profiling under storage and 1-MCP treatments showed both shared and divergent responses among selected *MsiJAZ* genes. Notably, *MsiJAZ1* was significantly repressed by storage but induced by 1-MCP, and was shown to physically interact with MsiPUB24, an E3 ubiquitin ligase involved in ethylene-mediated softening. These findings suggest that MsiJAZ1 may participate in a novel COI1-independent degradation pathway and mediate ethylene–JA crosstalk during fruit softening. Collectively, our results provide a comprehensive characterization of the JAZ family in *M. sieversii* and lay a solid foundation for further functional studies on JA signaling in apple fruit softening and quality improvement.

## Data Availability

The raw data supporting the conclusions of this article will be made available by the authors on request.

## Supplementary Materials

The following supporting information can be downloaded at: https://www.mdpi.com/article/10.3390/genes17070742/s1, Figure S1: Multiple sequence alignment of MsiJAZ proteins; Figure S2: Sequence logo of the conserved motifs in MsiJAZ proteins; Figure S3: Chromosomal distribution of *JAZ* genes in *M. sieversii*; Table S1: Genomic information of multiple species; Table S2: Primers used in this study; Table S3. Information of MsiJAZ genes and their encoded proteins identified in the *Malus sieversii* genome.
