# Supplementary material for: Genome-Wide Identification and Characterization of the JAZ Gene Family in Malus sieversii
Source: Genes (Basel). 2026 Jun 26;17(7):742. doi: 10.3390/genes17070742 (PMC13407427; doi:10.3390/genes17070742)
Supplement: Supplementary file 1 [file genes-17-00742-s001.zip › genes-4373167-supplementary.pdf]

Supplementary Figure S1. Multiple sequence alignment of MsiJAZ proteins.

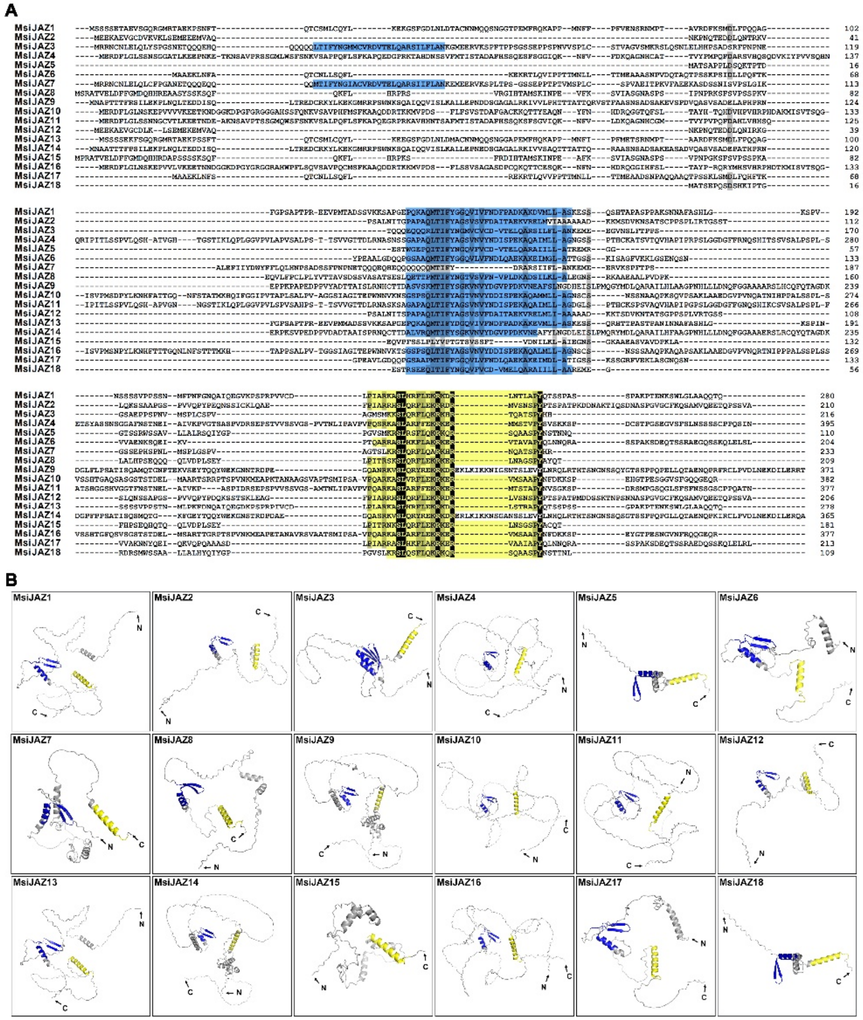

Supplementary Figure S2. Sequence logo of the conserved motifs in MsiJAZ proteins.

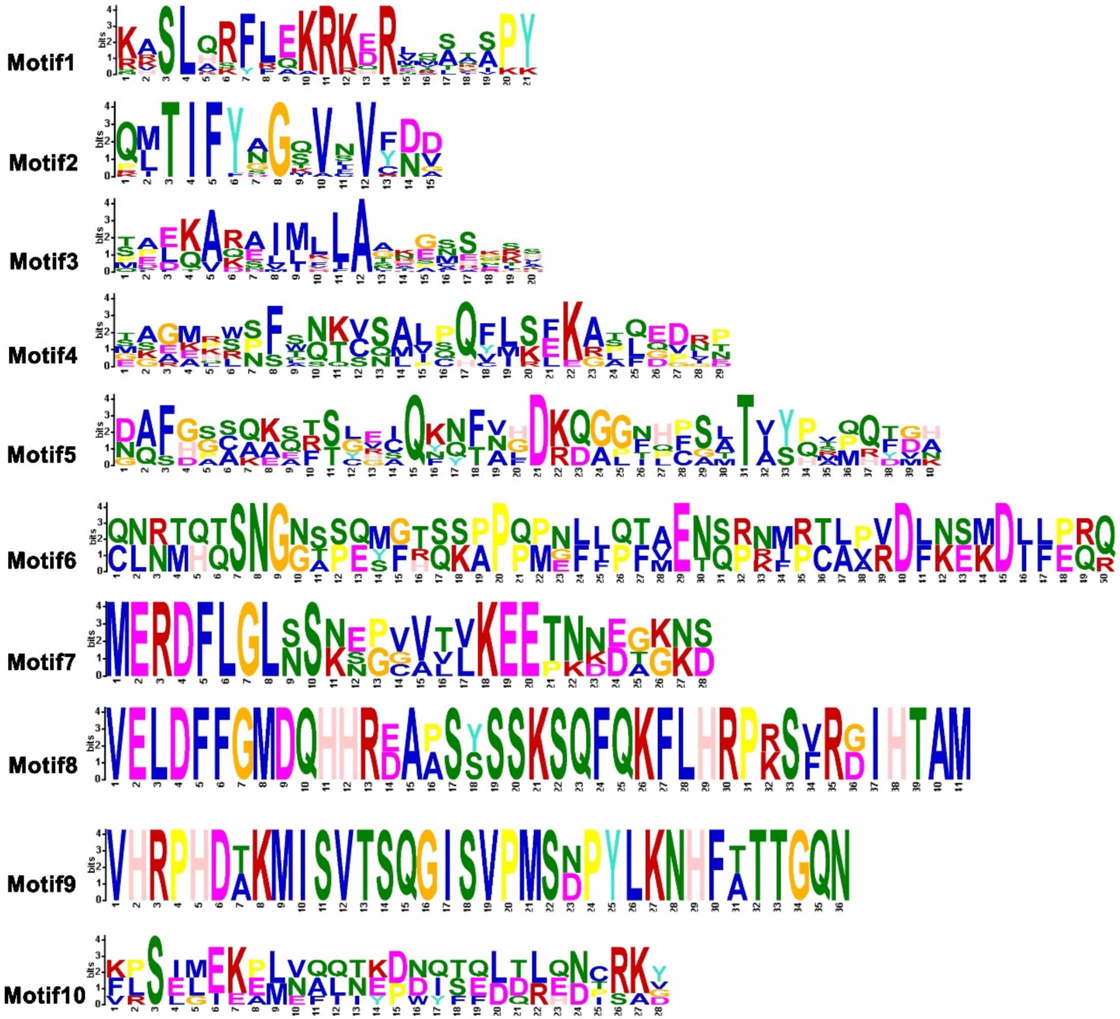

Supplementary Figure S3. Chromosomal distribution of *JAZ* genes in *M. sieversii*

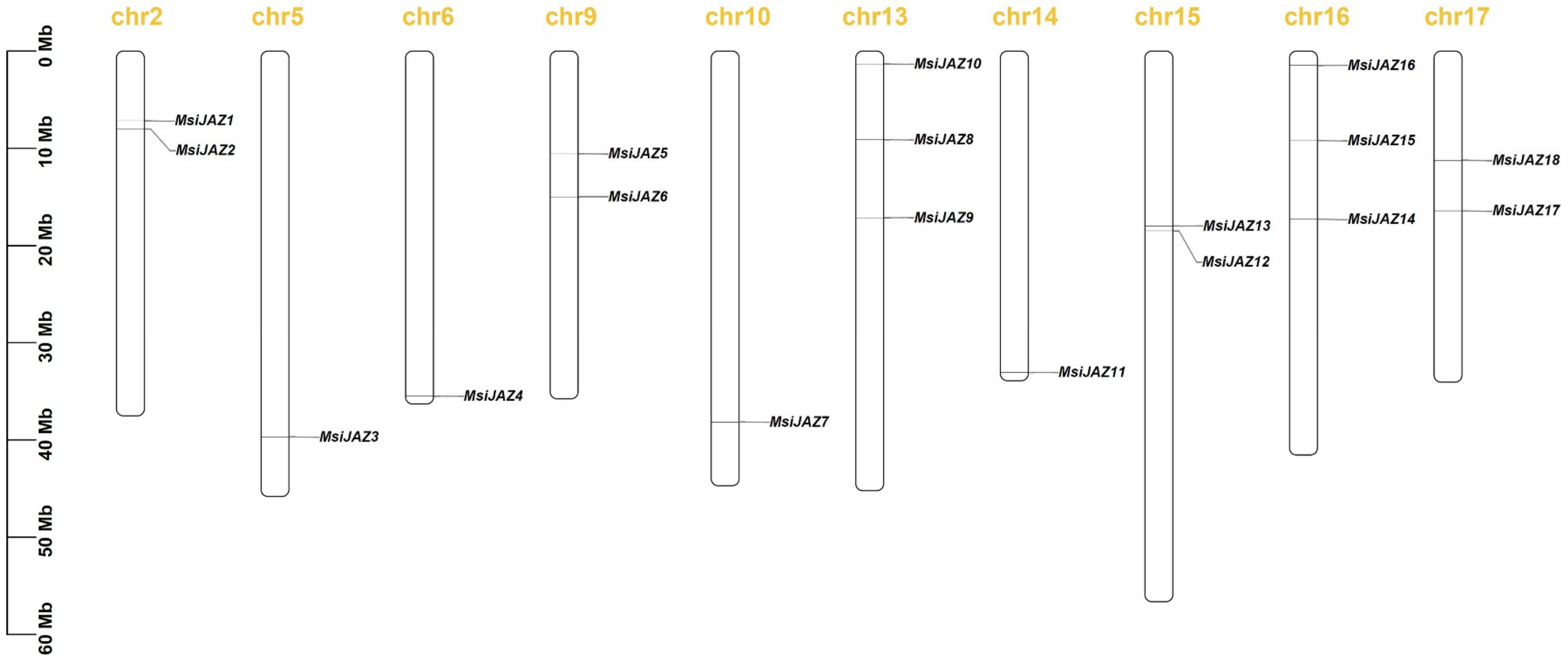

Supplementary Table S1. Genomic information of multiple species.

Species	Genome ID	Website
M. sieversii	v1.0	https://www.rosaceae.org/
A. thaliana	167	https://phytozome-next.jgi.doe.gov/
M. domestica	491	https://phytozome-next.jgi.doe.gov/
P. betulifolia	None	http://pyrusgdb.sdau.edu.cn/
V. vinifera	457	https://phytozome-next.jgi.doe.gov/
O. sativa	IRGSP-1.0	http://plants.ensembl.org/
S. lycopersicum	514	https://phytozome-next.jgi.doe.gov/

Supplementary Table S2. Primers used in this study.

Name	Sequence
MsiJAZ1-qPCR-F	GTCGAGTTCGTCGGAGACTG
MsiJAZ1-qPCR-R	TGAAGTTCGACGGCTTCTCC
MsiJAZ6-qPCR-F	ATGGGGTTGGCTACAACTGG
MsiJAZ6-qPCR-R	GTGCTACTGCTGTCACCCTT
MsiJAZ8-qPCR-F	AGAGCCTCCAGGAAACAACG
MsiJAZ8-qPCR-R	ATGCTATCCGCCTTGTCGAG
MsiJAZ11-qPCR-F	TATCCTGTGCCGCAGTTTGA
MsiJAZ11-qPCR-R	TCCTCCAAGTGGTTGCAGTT
MsiJAZ14-qPCR-F	TAAGCCTTCCAATGCAGCGA
MsiJAZ14-qPCR-R	CCCGCAGTCTGATATTGGCA
MsiJAZ15-qPCR-F	GCCTTCAAATCCGTCATCGC
MsiJAZ15-qPCR-R	TGTTATCCACCGTGAAAGAAGA
ACTIN-qPCR-F	ACACGGGGAGGTAGTGACAA
ACTIN-qPCR-R	CCTCCAATGGATCCTCGTTA
MsiPUB24-BD-F	GGAGGACCTGcatatgATGGATGATATTGAAGTTCCTCA
MsiPUB24-BD-R	GGATCCCCGGgaattcTCTGGGATACAGAGACATACCAA
MsiJAZ1-AD-F	AGATTACGCTcatatgATGTCGAGTTCGTCGGAGACTGC
MsiJAZ1-AD-R	CACCCGGGTGgaattcTTGGGTTTGCTGAGCAGCCAACC
MsiPUB24-nLUC-F	ACGAGCTCGGTACCCGggatccaATGGATGATATTGAAGTTCCTCA
MsiPUB24-nLUC-R	CGCGTACGAGATCTGgtcgacTCTGGGATACAGAGACATACCAA
MsiJAZ1-cLUC-F	GCGGTACCCGggatccaATGTCGAGTTCGTCGGAGACTGC
MsiJAZ1-cLUC-R	AACGAAAGCTctgcagTTGGGTTTGCTGAGCAGCCAACC

Supplementary Table S3. Information of *MsiJAZ* genes and their encoded proteins identified in the *Malus sieversii* genome.

Gene name	Gene ID	Protein size (aa)	MW (kDa)	pI	GRAVY	Localization prediction
MsiJAZ1	Msi_02g008620	280	30.29	9.26	-0.638	Nucleus
MsiJAZ2	Msi_02g009580	210	22.60	5.96	-0.515	Chloroplast
MsiJAZ3	Msi_05g026810	216	24.26	8.24	-0.645	Nucleus
MsiJAZ4	Msi_06g020180	395	41.47	9.11	-0.303	Nucleus
MsiJAZ5	Msi_09g012630	110	12.14	10.11	-0.480	Nucleus
MsiJAZ6	Msi_09g015960	204	22.10	9.43	-0.481	Chloroplast
MsiJAZ7	Msi_10g024320	233	26.45	6.23	-0.655	Nucleus
MsiJAZ8	Msi_13g011180	209	23.07	9.32	-0.367	Nucleus
MsiJAZ9	Msi_13g017150	371	41.21	8.22	-0.761	Nucleus
MsiJAZ10	Msi_13g001740	382	40.56	9.15	-0.510	Nucleus
MsiJAZ11	Msi_14g023140	377	39.40	9.19	-0.230	Nucleus
MsiJAZ12	Msi_15g020860	206	22.00	6.61	-0.595	Nucleus
MsiJAZ13	Msi_15g020450	278	30.39	9.00	-0.685	Nucleus
MsiJAZ14	Msi_16g017840	365	40.25	7.64	-0.817	Nucleus
MsiJAZ15	Msi_16g011660	181	19.86	9.41	-0.529	Nucleus
MsiJAZ16	Msi_16g002010	377	40.47	9.34	-0.602	Nucleus
MsiJAZ17	Msi_17g014780	213	23.25	9.38	-0.489	Chloroplast
MsiJAZ18	Msi_17g011810	109	12.22	8.89	-0.442	Nucleus
